# Multi-Parametric Analysis of Reliability and Energy Consumption in IoT: A Deep Learning Approach

**DOI:** 10.3390/s19020309

**Published:** 2019-01-14

**Authors:** Muhammad Ateeq, Farruh Ishmanov, Muhammad Khalil Afzal, Muhammad Naeem

**Affiliations:** 1 Department of Computer Science, COMSATS University Islamabad, Wah Campus, Wah Cantt 47040, Pakistan; rmateeq@yahoo.com; 2 Department of Electronics & Communication Engineering, Kwangwoon University, Seoul 01897, Korea; 3 Department of Electrical & Computer Engineering, COMSATS University Islamabad, Wah Campus, Wah Cantt 47040, Pakistan; muhammadnaeem@gmail.com

**Keywords:** IEEE 802.15.4, packet delivery ratio, energy consumption, prediction, deep learning, internet of things, wireless sensor networks

## Abstract

Small-to-medium scale smart buildings are an important part of the Internet of Things (IoT). Wireless Sensor Networks (WSNs) are the major enabler for smart control in such environments. Reliability is among the key performance requirements for many loss-sensitive IoT and WSN applications, while Energy Consumption (*EC*) remains a primary concern in WSN design. Error-prone links, traffic intense applications, and limited physical resources make it challenging to meet these service goals—not only that these performance metrics often conflict with one another, but also require solving optimization problems, which are intrinsically NP-hard. Correctly forecasting Packet Delivery Ratio (*PDR*) and *EC* can play a significant role in different loss-sensitive application environments. With the ever-increasing availability of performance data, data-driven techniques are becoming popular in such settings. It is observed that a number of communication parameters like transmission power, packet size, etc., influence metrics like *PDR* and *EC* in diverse ways. In this work, different regression models including linear, gradient boosting, random forest, and deep learning are used for the purpose of predicting both *PDR* and *EC* based on such communication parameters. To evaluate the performance, a public dataset of the IEEE 802.15.4 network, containing measurements against more than 48,000 combinations of parameter configurations, is used. Results are evaluated using root mean square error and it turns out that deep learning achieves up to 98% accuracy for both *PDR* and *EC* predictions. These prediction results can help configure communication parameters taking into account the performance goals.

## 1. Introduction

Wireless communication and sensing are major enablers for Wireless Sensor Networks (WSNs) and the Internet of Things (IoT). While considering small-to-medium scale buildings, WSNs [1] are placed at the very heart to facilitate smart operations [2,3]. Some of the prominent application areas of WSN-based IoT for smart buildings include energy and water management, fire and smoke detection, appliance and light control, security and surveillance, and entertainment [2,4]. IEEE 802.15.4 is one of the most popular communication standards used in WSNs. According to a recent survey, WSN deployments for which communication standards are known, more than 50% are based on IEEE 802.15.4 standards [5].

Depending on the domain of deployment and application scenarios, WSNs and IoT have different service requirements to meet including energy, reliability, delay, and Throughput (THP) [6,7]. Recently, there has been a tremendous research effort to improve all possible performance dimensions of WSNs and IoT [6,8]. Optimizing multiple metrics is frequently modeled as an optimization problem that is often NP-hard [9]. Optimizing one metric also tends to conflict with the other metrics, and a trade-off is needed. Mathematical programming based serialization methods and nature-inspired meta-heuristic algorithms are popular choices in this context [9]. Such methods workaround NP-hardness, compromising on accuracy. In addition, adaptivity is considered a mandatory trait for all modern communication systems. Hence, there is a growing trend of using data-driven approaches and Machine Learning (ML) [10,11,12] to meet the performance challenges faced by WSNs and IoT. A case study based approach to facilitate intelligence is presented in Ref. [13]. In summary, the factors that motivate the current study include:

- Diversified QoS requirements in ever-evolving WSN and IoT infrastructure.
- Intrinsic NP-hardness of the optimization problems to facilitate multi-objective and conflicting QoS goals.
- Communication infrastructure that makes it viable for large-scale performance data to become available, and
- Availability of sophisticated, robust, and practical deep learning algorithms that can learn from data and promise adaptivity with near optimal accuracy.

The focus in this paper is predicting reliability in the form of Packet Delivery Ratio (*PDR*) and Energy Consumption (*EC*) in IEEE 802.14.5 based networks using Deep Learning (*DL*). It is argued that various communication parameters (e.g., Transmission Power (*TP*), Packet Size (*PS*), Queue Size (*QS*), Maximum Transmissions (*MT*), and inter-node Distance (*DT*), etc.) play a vital role in defining PDR and EC. Therefore, it is of primary importance to understand the dynamics of these metrics in relation to a detailed stack parameter configurations. Findings from this study can help extend the research effort encompassing a wider range of parameters including even protocols at different layers. In this paper, an adaptive system to predict PDR and EC based on different stack parameter configurations is proposed. The system used performance data and applied deep learning to make data-driven predictions for both PDR and EC. In particular, the suggested design achieves an adequate prediction accuracy without having to solve any intractable optimization problem. To the best of our knowledge, this is the first work that applies deep learning to predict PDR and EC based on real data from the IEEE 802.15.4 network. The main contributions of this work can be summarized as follows:

- Analysis of the relationship between PDR and EC in relation to more than 48,000 stacks of parameter configurations.
- Development and evaluation of a deep learning model for predicting PDR and EC. It is elaborated that the deep learning model, with a suitable set of parameters, can be implemented on a well-equipped remote server, thus facilitating rich learning results by use of sophisticated algorithms trained on large, growing and diversifying performance data.
- Keeping the resource-constrained user-devices free from the computational load by making the prediction data available to the user premises in the form of metric:value, and parameter:values pairs. This data can be directly used for choosing values of communication parameters, meeting the constraint for metrics under consideration.
- Desirably accurate estimation of performance metrics without having to deal with the optimization problems which are intrinsically NP-hard.
- A flexible and evolving system that can adapt to the circumstantial and even design changes that may occur over time. This adaptiveness can facilitate a sustainable system in contrast to the most client-side approaches where learning is based on the missing value prediction in the output matrix rather than the input communication parameters.

The rest of this paper is organized as follows: a literature survey is carried out in Section 2. Description of data and deep learning models used are discussed in Section 3. Prediction results are presented in Section 4. Section 5 concludes the paper.

## 2. Related Work

With energy as a fundamental design focus, a number of application areas are identified to be loss-sensitive and mission-critical (e.g., surveillance, disaster recovery, security, environmental monitoring, emergency/rescue, and event-driven applications) [14]. Efforts to improve reliability and energy have been carried out in the form of protocols at different layers of the network stack, and cross-layer approaches have also been proposed [6,14]. However, there is a growing interest in designing adaptive systems that can learn from the ever-changing circumstances and adjust accordingly without having to solve intractable optimization problems. In this context, there is an inherent interaction and inter-dependence between IoT and cloud services. To facilitate software level adaptation, a meta-data layer is proposed in Ref. [15] for web services. In Ref. [16], a trusted third party based scheme is proposed to improve quality of experience in vehicular cloud services. A network slicing scheme based on QoS requirements is proposed in Ref. [17] for dense vehicular clouds. A big data based framework to facilitate life care-aware decision-making is presented in Ref. [18]. A summary of the literature related to predictions is presented in Table 1. The discussed literature is divided into two categories, namely, client-side and server-side prediction approaches. In addition to the objective, inputs, outputs, learning algorithms, evaluation methods, the domain of application, and sources of datasets used for experimentation are listed in Table 1. In the following, prediction approaches are narrated, divided into two categories.

### 2.1. Client-Side Predictions

Client-side approaches make use of the live performance data on the end-devices to make intelligent service choices. With the benefit of live decision-making comes the drawback of the limited capacity of IoT devices, thus compromising on the quality of learning as well as overloading an already constrained device. In such settings, collaborative approaches like Matrix Factorization (MF) are used to predict missing values in the Quality of Service (QoS) vectors of various services. Some of the main contributions include [8,21,23,26,27,30]. All these works predict missing values for Response Time (RT) and THP, under various matrix densities and dimensionalities of learning algorithms. MF is used in Refs. [21,23], whereas Ref. [27] proposes long short-term memory for the same purpose. A Pearson’s correlation coefficient and Kendal’s tau based collaborative approach are adopted in Ref. [8]. A context-sensitive MF technique is proposed in Ref. [26], whereas [30] uses a deep neural model. All these works are evaluated using standard regression metrics and most of these use an established dataset released by Zheng [22] containing the RT and THP of 339 users and 5825 services.

### 2.2. Server-Side Predictions

Server-side approaches put the load of computation on a remote server with end-devices primarily utilizing the recommendations from the server. Having adequate processing capacity, sophisticated learning algorithms can be applied to large-scale performance data. Thus, the potential to achieve good results is intrinsically high. Although the computational burden is eased, a certain amount of periodic communication overhead has to be afforded in such settings. Nevertheless, considering the limitations of IoT devices, such an overhead, if controlled properly, can be reasonably justified. Some of the main server-side prediction approaches are presented in Refs. [19,24,32]. Tao [19] used packet reception ratio, Received Signal Strength Indicator (RSSI), Signal-to-Noise Ratio (SNR) and Link Quality Indicator (LQI) to predict the probability of delivery of the next packet. Importantly, RSSI, SNR, and LQI are all receiver side values that are used to determine the success probability of next packet delivery at the transmitter. Neural Networks (NN) based Packet Loss Ratio (PLR) prediction results are presented in Ref. [24]. The work mentioned [24] used inter-packet interval, number of nodes, received Packets (rP) and erroneous Packets (errP) as input features. It is interesting to notice that rP and errP are directly used as features for predicting PLR. Ayhan [32] used neural networks to predict TP level in relation to network Lifetime (LT) and inter-node DT. In order to predict one of these metrics, two others were taken as features in this work [32].

The client-side approaches are limited to missing value predictions from the matrix of values for the metric under consideration. This limits the quality and applicability of prediction models to the diverse application scenarios and heterogeneous communication settings present in the IoT. It is argued that a model capturing the variations in different communication, circumstantial and application specific variables, affecting the QoS, can better prepare a model for sustainable learning and potentially yielding more accurate predictions. The amount of resources required for this kind of learning cannot be enabled on sensor and IoT devices with limited capabilities. The server-side approaches, on the other hand, do not take into account a wider set of parameters and often use one predicted metric as a feature to predict another metric (e.g., in Ref. [32], while predicting any of LT, TP or DT, the other two are used as input features to the learning model), or the values directly translating into the metric of interest are used as input features (e.g., in Ref. [24] rP and errP are used to predict PLR). The realization of such a system is far from being practical. In summary, the common limitations of all these efforts are that (i) the datasets used are confined to a few parameters only and did not grasp the diversity of wider parameter configurations; thus, they lacked in capturing the important relationship between configurable parameters and relevant performance metrics, and (ii) did not consider energy consumption.

## 3. System Model

### 3.1. Overview

**Figure 1:**
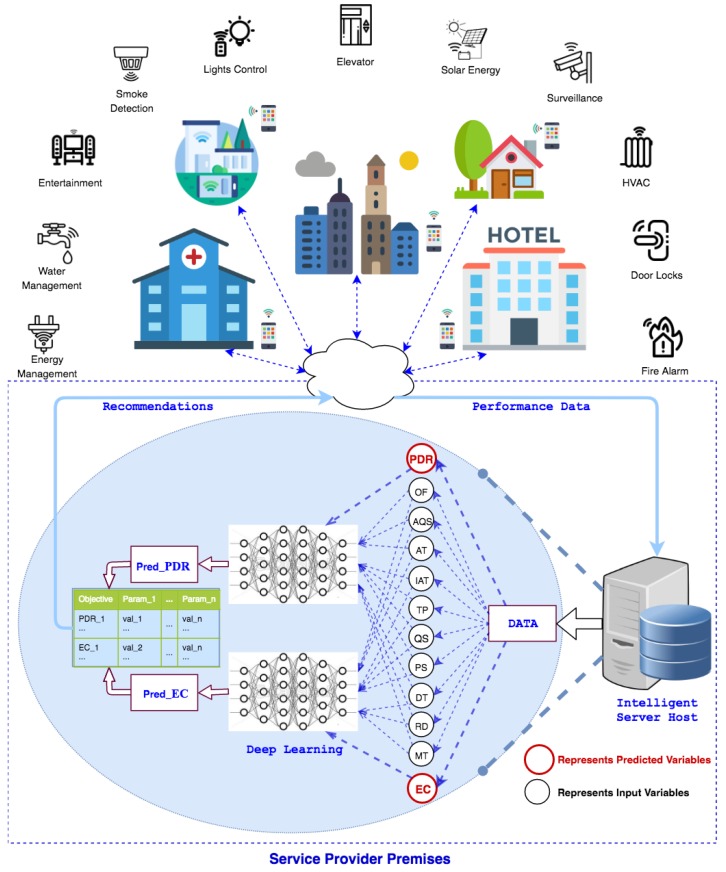
System model.

In the proposed work, an IoT enabled IEEE 802.15.4 network for small-to-medium scale buildings is considered (Figure 1). Each building has smart facilities (e.g., energy management, door locks, heat ventilation air-conditioning, security and surveillance, lights, entertainment, and water management) with a central control within the premises which communicates with all smart installations for management operations and control. In addition to regular communication, the performance data consisting of parameters and metrics of interest is periodically transmitted from the site of deployment to the service provider through this central control. A server collects this data and runs deep learning algorithms to identify the relationships between performance metrics of interest (PDR and EC in this case) and communication parameters (TP,DT,PS, etc.), as highlighted in Figure 1. This learning process keeps adapting as different aspects of the network evolve (e.g, change in; QoS requirements, communication parameters including interference, size, and dimensions of the network, and channel quality, etc.), and new data becomes available. Against each performance metric constraint, a set of recommended values for the communication parameters that meet (maximize/minimize) the required goals for the metric are sent to the central controller within the consumer premises. This information is sent in the form of a table as shown in Figure 1. This table contains a set of values for each metric and recommended values for a list of relevant parameters that may help meet the constraint for the metric under consideration. Thus, for the end-device, it is a simple table lookup operation. The controller at the user site uses this information to select the suitable values for the parameters considering performance constraints.

### 3.2. The Data

We have used a publicly available dataset, collected over a period of six months, in the IEEE 802.15.4 network [33]. In the experiments, more than 48,000 configurations of seven key stack parameters were used. At the physical layer, parameters used are: *DT* between nodes and *TP level*. At the Medium Access Control (MAC) layer, parameters are: *MT*, *Retry Delay* (RD), and *maximum*QS of the packets waiting at MAC layer. At the application layer, parameters are: *packet Inter-Arrival Time* (IAT), and *PS*. In addition to these stack parameters, rich per-packet meta-data was collected including: *buffer OverFlow* (OF), *Actual Queue Size* (AQS), and *Actual Transmissions* (AT). A list of abbreviations and symbols is provided in Table 2 and Table 3 summarizes these parameters along with their explanation and the range of values used in the experiments. The values for performance metrics, such as PDR and EC, were calculated using:

(1) $$PDR=\frac{Pkts_{acked}}{Pkts_{tot}},$$

(2) $$EC=\frac{power\times time\times (D+H)}{D\times PDR_{T}},$$

where,

(3) $$PDR_{T}=\frac{Pkts_{acked}}{Pkts_{att}},$$

and *time* is 0.004 ms for transmission rate of 250 Kbps, *power* is taken according to the data-sheet of CC2420, and *D* and *H* represent packet payload size and stack overhead size, respectively.

#### Key Observations

**Figure 2:**
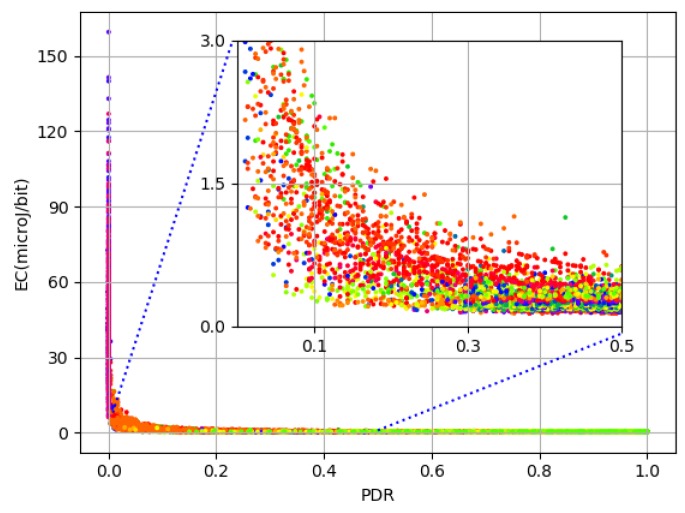
Relationship between PDR and EC.

**Figure 3:**
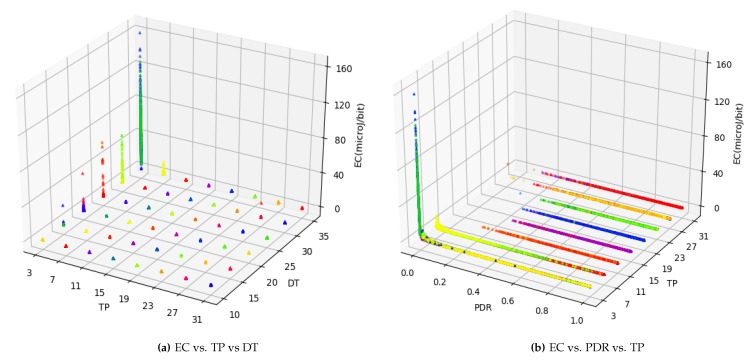
EC in relation to PDR,TP and DT.

The relationship between PDR and EC is shown in Figure 2. Being a ratio, PDR ranges between 0 and 1 with median (0.97) close to 1. Both PDR and EC appear to lie in close proximity but for the lower quarter of PDR values. A zoomed inner frame in Figure 2 reflects this relationship for the bottom half of PDR values. It is evident that there is a rapid hike in EC as PDR falls below 0.3. This tremendous variation in EC values can be explained in two ways. First, as losses (radio) increase, energy consumed to transmit for the lost frame gets wasted and thus induces a rise in EC/bit. Second, although the total number of packets transmitted is 300, depending on the maximum value of MT (which can be either 1, 3, or 5), the actual number of attempts can be as high as 1500. This phenomenon leads to an enormous positive skew with a very high standard deviation of 7.31 compared to both mean (1.30) and median (0.27) for EC values. Considering the forward error correction limit of $10^{-3}$ on bit error rate, which is expected to be far less in modern wireless communication systems, a PDR of less than 25% itself remains under question for performance characterization. In order to further understand the parameters working behind the scenes, Figure 3a shows a 3D plot of EC in relation to TP and DT. It is evident that EC is extremely high when the TP level is at a minimum (i.e., 3). This is due to the fact that the TP level is not adequate for successful transmission and, as a result, frames suffer radio losses. This results in increased EC for successfully transmitted data bits. However, the values for EC keep decreasing as the DT decreases. To understand more, Figure 3b shows both EC and PDR in relation to TP. It is again clear from the plot that, for a TP level of 3, and low PDR, there is a visible hike in EC. Despite this large deviation, in order to consider the entire amount of data, better prepare it for learning, and expect a practical prediction accuracy, these statistics encourage us to split the EC data based on PDR values. Median based z-score measure is used to decide the split because PDR has a negative skew with median (0.97) being higher than mean (0.87). The formula used to split EC data is:

(4) $$z_{scroe}=PDR_{med}\pm 3\times PDR_{std}.$$

This results in 0.25 as a value for $z_{scroe}$, where $PDR_{med}$ is 0.97 and $PDR_{std}$ is 0.24, and, based on this value, the data is split into two parts for prediction of EC; first, where PDR>=0.25 and the second where PDR<0.25.

The first part consists of 95.1% data (referred to as ECdense) ranging between 0.136 and 1.155 with both mean (0.279) and median (0.267) closely located and a small standard deviation of 0.087. This reflects an acceptable distribution for ECdense improving the chances of good prediction accuracy. The data in the second split (referred to as ECsparse) comprises only 4.9% of the total data spread over the range of 0.161 and 159.453 and are seen as outliers. Mean (26.085) and standard deviation (26.06) are close enough for ECsparse data. However, the median (4.166) is still relatively low, thus indicating dispersion of data. As a result, before proceeding to deep learning, the data have three target variables to predict: PDR, ECdense, and ECsparse.

### 3.3. Deep Learning

Deep learning is employed for modeling the relationships between communication parameters and performance metrics. The aim is to predict PDR, ECdense, and ECsparse (referred to as target outputs hereafter) based on more than 48,000 combinations of seven pre-configured and three per-packet input variables (referred to as features hereafter). The data is represented in the form of features, and target outputs as: ($f_{m,1},f_{m,2},\ldots ,f_{m,10},PDR,EC$). Here, *m* represents the total number of tuples in the data which are 48,384 to be exact. In summary, three deep leaning models are trained for three target outputs with ten input features. For training and evaluating the deep learning models, data are split into training (50%), validation (20%), and test (30%) sets. The following hyper-parameters are tuned for the neural network: the number of dense layers used is 10, the learning rate (α) is set to 0.001, the maximum number of epochs used is 500 while the training process was stopped if the model did not improve for 150 consecutive iterations. As an exception, for ECsparse, the number of epochs used is 1500. This is because models kept improving for longer periods due to highly deviant data. It is worth mentioning that the values for all these deep learning parameters (including learning rate, number of layers, and epochs) were chosen empirically. In the following, the computation used for the neural networks consisting of two passes (forward and backward) is described. A forward pass is computed as:

(5) $$Z^{{}^{\left[l\right]}}=W^{{}^{\left[l\right]}}\cdot A^{{}^{\left[l-1\right]}}+b^{{}^{\left[l\right]}},$$

(6) $$A^{{}^{\left[l\right]}}=g^{{}^{\left[l\right]}}\left(Z^{{}^{\left[l\right]}}\right),$$

where *l* represents layer number. Z,W,b and *A* represent output vector for the activation function, weights vector used for features, bias/parameter, and the input vector at a layer, respectively. Rectified linear unit is used as the activation function and is represented by *g*. A backward pass which is responsible for computing rate of change through derivatives with an aim to update weights is calculated as:

(7) $$dZ^{{}^{\left[l\right]}}=dA^{{}^{\left[l\right]}}\times g^{{}^{{\left[l\right]}^{{}^{\prime }}}}\left(Z^{{}^{\left[l\right]}}\right),$$

(8) $$dW^{{}^{\left[l\right]}}=\frac{1}{m}\left(dZ^{{}^{\left[l\right]}}\cdot A^{{}^{[l].T}}\right),$$

(9) $$db^{{}^{\left[l\right]}}=\frac{1}{m}\sum_{i=1}^{m}Z^{{}^{\left[l\right]}},$$

(10) $$dA^{{}^{\left[l-1\right]}}=W^{{}^{[l].T}}\cdot dZ^{{}^{\left[l\right]}},$$

where $g^{{}^{\prime }}$ represents the derivative of the activation function, and *m* is the number of tuples in the training data. Gradient descent algorithm is run with an objective to minimize the error on the validation set updating *W* and *b* until convergence, using:

(11) $$W^{{}^{\left[l\right]}}=W^{{}^{\left[l\right]}}-\alpha \times dW^{{}^{\left[l\right]}},$$

(12) $$b^{{}^{\left[l\right]}}=b^{{}^{\left[l\right]}}-\alpha \times db^{{}^{\left[l\right]}}.$$

#### Model Evaluation

In order to evaluate the accuracy of deep learning model, the prediction error is calculated as:

(13) $$err_{k}=Y_{k}^{actual}-Y_{k}^{predicted},$$

where *k* represents the index of a tuple, and $Y_{k}^{actual}$ and $Y_{k}^{predicted}$ represent actual and predicted values for $k^{th}$ tuple in training data. This $err_{k}$ is used to compute the overall Root Mean Squared Error (*RMSE*), Mean Percentage Error (*MPE*), and Mean Absolute Percentage Error (*MAPE*):

(14) $$RMSE=\sqrt{\frac{\sum_{k=1}^{m_{t}}{\left(err_{k}\right)}^{2}}{m_{t}}},$$

(15) $$MPE=\frac{100}{m_{t}}\sum_{k=1}^{m_{t}}\frac{err_{k}}{Y_{k}^{actual}},$$

(16) $$MAPE=\frac{100}{m_{t}}\sum_{k=1}^{m_{t}}\left|\frac{err_{k}}{Y_{k}^{actual}}\right|,$$

where $m_{t}$ represent the number of examples in the test data. The RMSE computes the sum of the squares of differences of prediction errors on all data points divided by the number of samples, and their square root is taken to get the normalized figure representative of the range of values of predicted output. MPE represents the prediction error as a percentage of the actual value, whereas MAPE rules out any balancing effect due to the cancellation of positive and negative errors.

The procedure of the proposed system model is presented in Algorithm 1. The input data comes from the user premises in the form of various communication parameters and QoS targets. After necessary steps, deep learning models are trained for each QoS target (steps 4 to 12). Having trained the model to a sufficient accuracy, the recommendations are transmitted back to the user premises in the form of target:value and parameter:value pairs.
**Algorithm 1:** The deep learning based procedure to predict PDR and EC
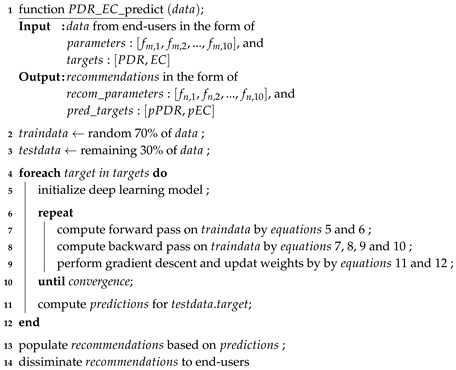


## 4. Results and Discussion

In this section, prediction results for both PDR and EC are described. The prediction error for all values in test data is shown in the form of scatter plots. RMSE for each individual feature and the best of their combinations of all lengths is presented. In addition, to better characterize the error, histograms of percentage error are plotted.

### 4.1. Packet Delivery Ratio

**Figure 4:**
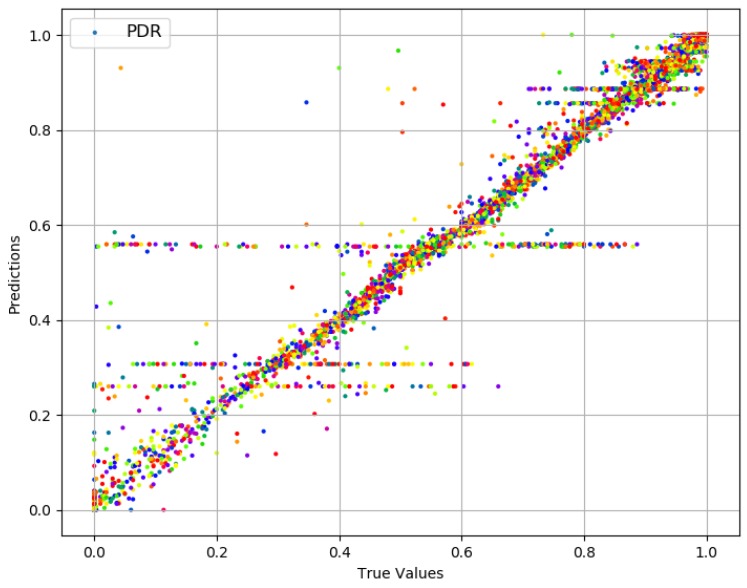
Actual values vs. predictions for PDR.

**Figure 5:**
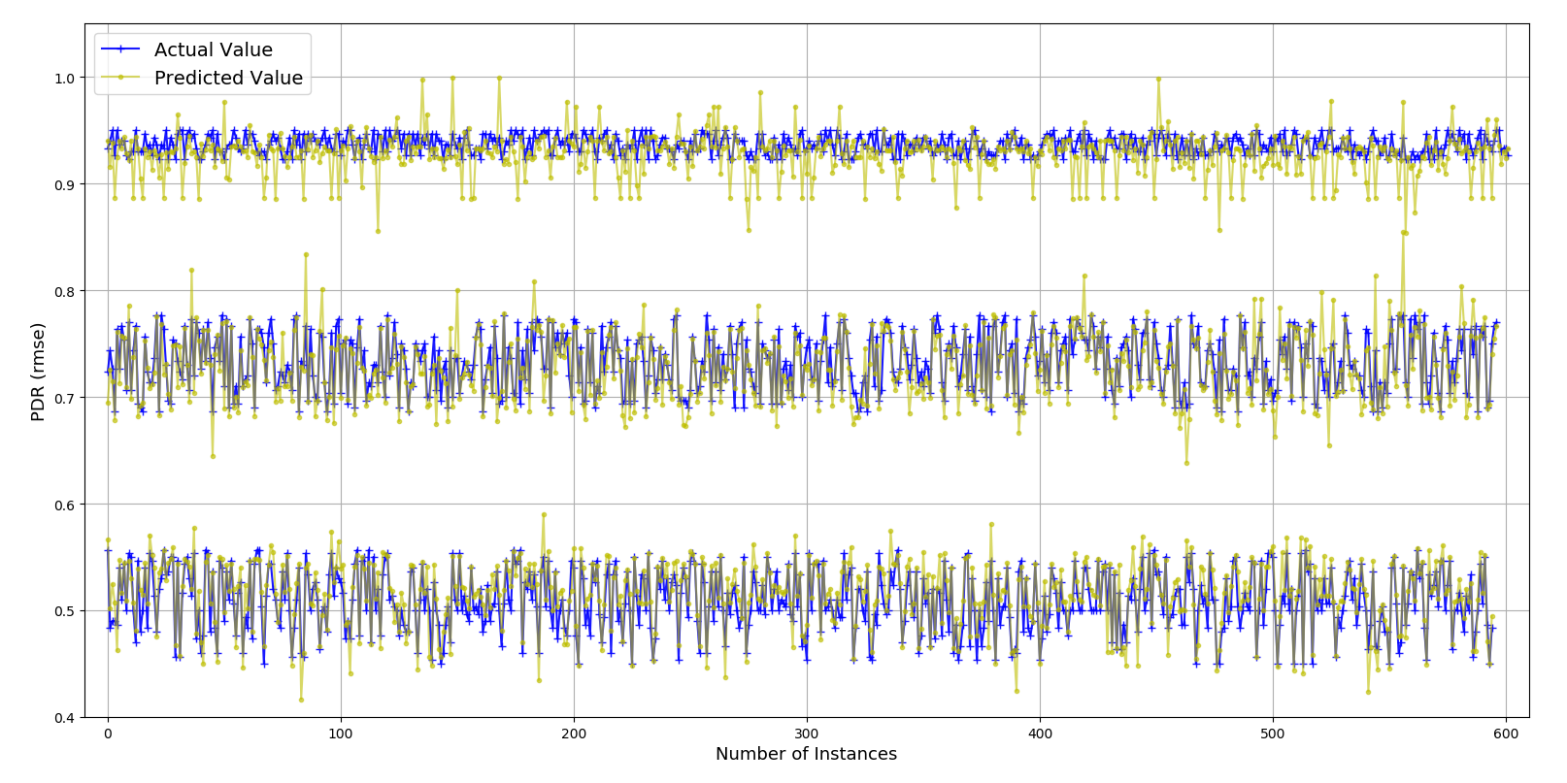
Actual values vs. predictions for PDR (selected data).

**Figure 6:**
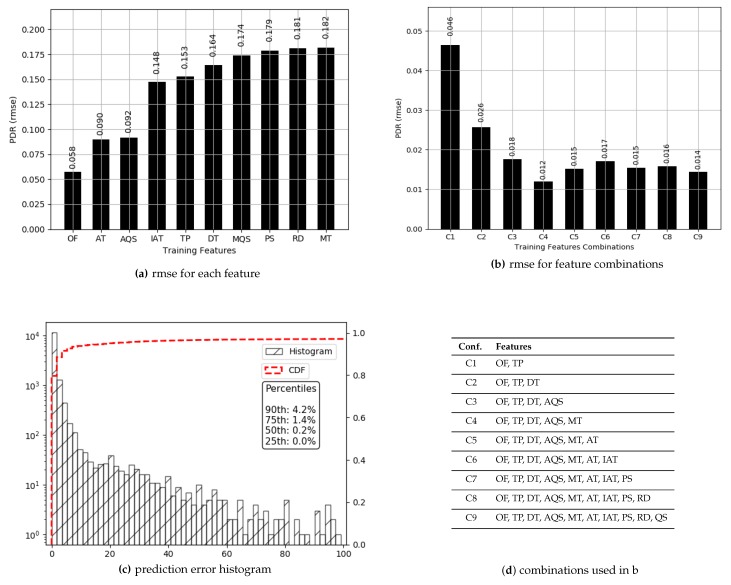
Prediction results for *PDR*.

The values for PDR have a high median (0.97), and 76% of those are above 0.80. Prediction results for PDR are presented in Figure 4. True values and predicted values are plotted which lie in a close proximity to the ideal diagonal line. To have a closer look, predicted values for three portions of selected data are plotted against actual values in Figure 5 from three different ranges (0.44 and 0.57, 0.68 and 0.78, 0.93 and 0.94). It is evident that the range shrinks for the same number of data points as PDR gets higher. It is also visible from the plots in Figure 5 that the predicted values are highly aligned with the actual values, in general. In order to better understand the prediction performance, further results are revealed in Figure 6. RMSE for individual features are shown in Figure 6a, where OF, AT, and AQS yield better prediction accuracy compared to other pre-configured features. This is because OF directly translates into queuing losses with AQS as an implicit indicator for the potential of the same, whereas AT directly defines the other (primarily radio) losses that occurred. Therefore, OF and AT produce a minimum error because not only do they directly define losses but they also have fine-grained per-packet values compared to the values for pre-configured features. Results for the combination of features are presented in Figure 6b, where five features, namely: OF, AT, TP, DT and MT, result in a minimum RMSE of 0.012 achieving the overall best prediction accuracy of 98.8% over the range of PDR values. The *x*-axis label codes, used in Figure 6b, are listed in Figure 6d. Finally, the prediction error is characterized in Figure 6d, where 90% of the predictions have an error of 4.2% or less of the actual PDR values. It is important to note that the addition of any further features after OF, AT, TP, DT, and RMSE does not result in any significant improvement in the accuracy.

### 4.2. Energy Consumption

Based on splits made in the EC data, prediction results are separately presented for ECdense and ECsparse.

#### 4.2.1. Dense Data

**Figure 7:**
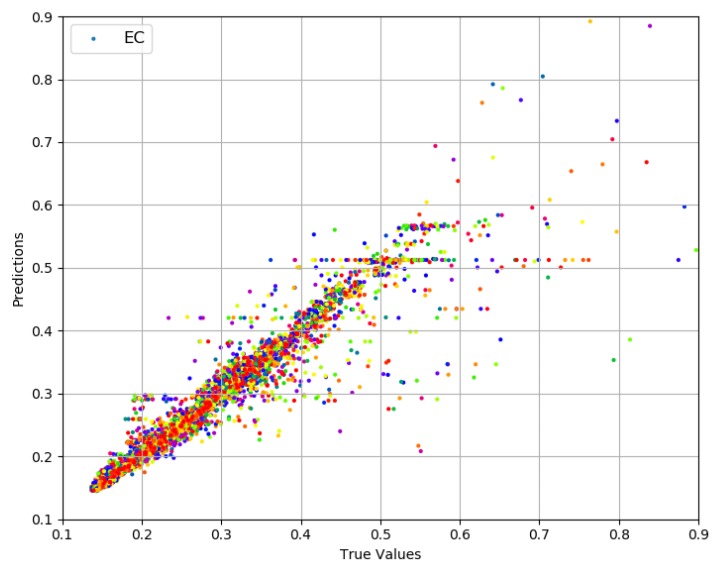
Actual values vs. predictions for ECdense.

**Figure 8:**
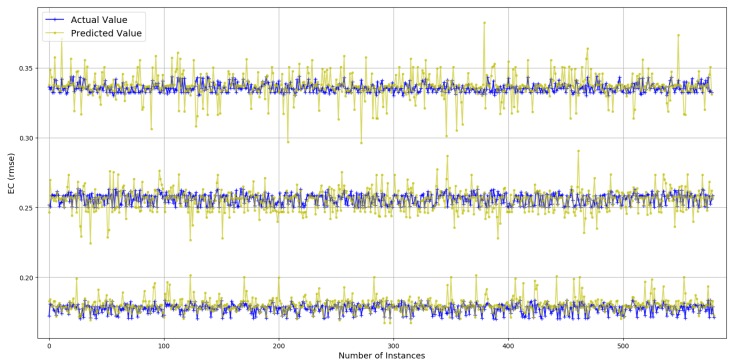
Actual values vs. predictions for ECdense (selected data).

**Figure 9:**
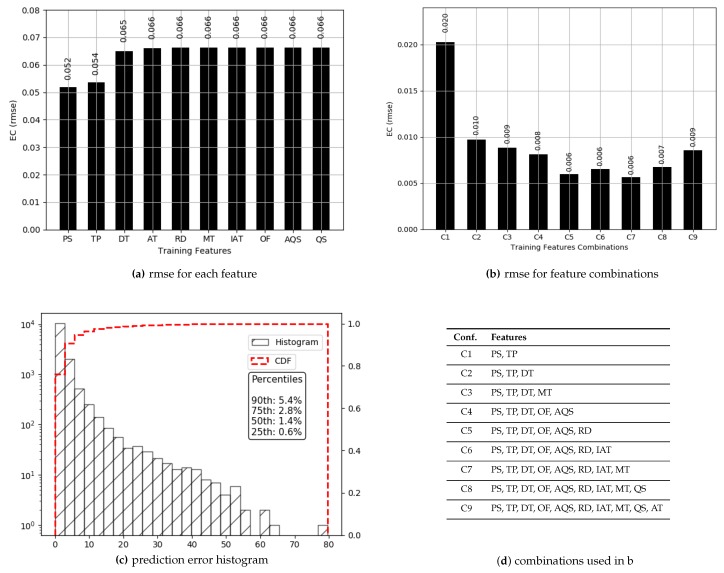
Prediction results for ECdense.

The values for ECdense range between 0.13 and 1.15 with more than 98% of the values less than 0.5, thus offering a range of 0.37. Prediction results are presented in Figure 7. True values and predicted values are plotted which lie in a close proximity to the ideal diagonal line. Most of the data lie between 0.13 and 0.5, and the prediction results do not diverge a lot from the expected diagonal. To have a closer look, predicted values for three portions of selected data are plotted against actual values in Figure 8 from three different ranges (0.170 and 0.181, 0.244 and 0.256, 0.331 and 0.342). These ranges indicate an even spread of data. It is visible from the plots in Figure 8 that the predicted values are highly aligned with the actual values, in general. More detailed prediction results are discussed in Figure 9. According to Figure 9a, PS and TP are two of the best features resulting in a lesser RMSE than others. The rest of the features, individually, achieve almost the same prediction accuracy. Overall RMSE reaches its minimum (0.006) with six features. Figure 9b consists of OF, AT, TP, PS, DT, and RD, yielding an accuracy of more than 98% even if a close range of max (0.5) − min (0.13) is considered. However, the improvement after three features (TP, PS and DT) is only 1.1%. Therefore, it can be concluded that TP, PS and DT are the most important and contributing features for capturing the variations in predictions for ECdense data. It makes a lot of sense, as TP directly defines the amount of power used for transmissions, and PS plays a decisive role because varying sizes change the proportion of control data that directly influence the amount of energy used per data bit. The third most important feature, DT, in combination with TP, influences the radio success probability, which is also very important in defining the EC/bit. The *x*-axis label codes used in Figure 9b are explained in Figure 9d. The prediction error is characterized in Figure 9d, where 90% of the predictions have an error of 5.4% or less of the actual ECdense values.

#### 4.2.2. Sparse Data

There are very limited values in the ECsparse data comprised of 4.9% of the total data and this portion is treated as an outlier. However, compared to the unsplitted data, descriptive statistics are expected to be more normal; however, there is still a notable skew with high values for the mean (21.09) and standard deviation (26.07) and a comparatively low median (4.16). The 10th, 25th, 50th, 75th, and 90th percentiles are 0.509, 0.917, 4.166, 40.96 and 57.11, respectively. This indicates the progression in the values for ECsparse. Despite deviant data, a deep learning model is trained to seek prediction results for ECsparse.

**Figure 10:**
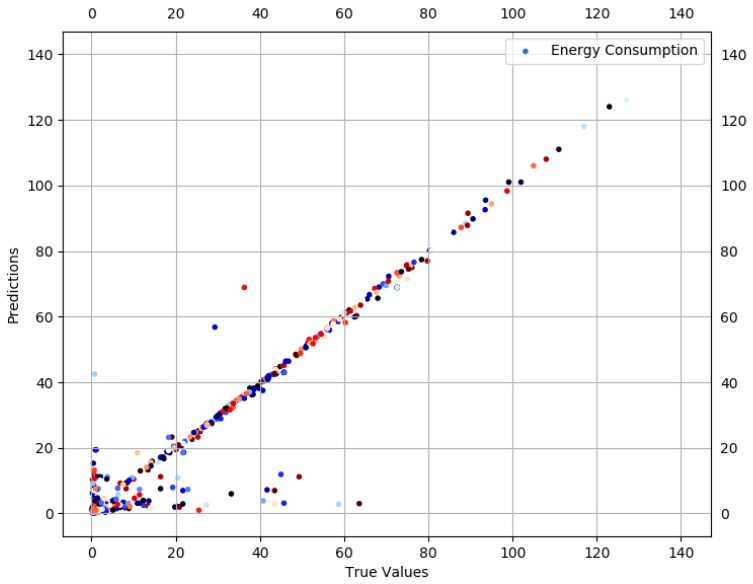
Actual values vs. predictions for ECsparse.

**Figure 11:**
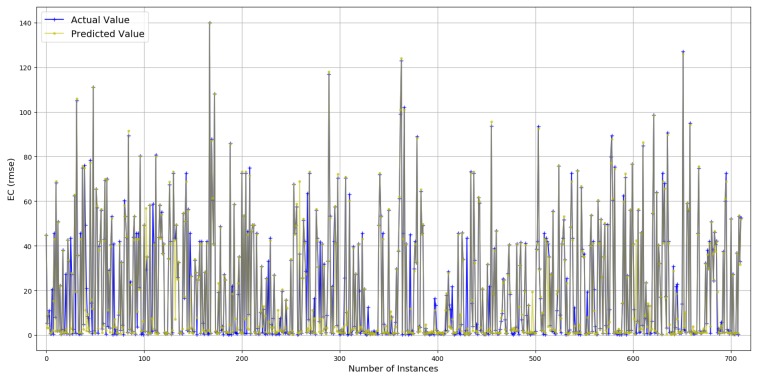
Actual values vs. predictions for ECsparse (selected data).

**Figure 12:**
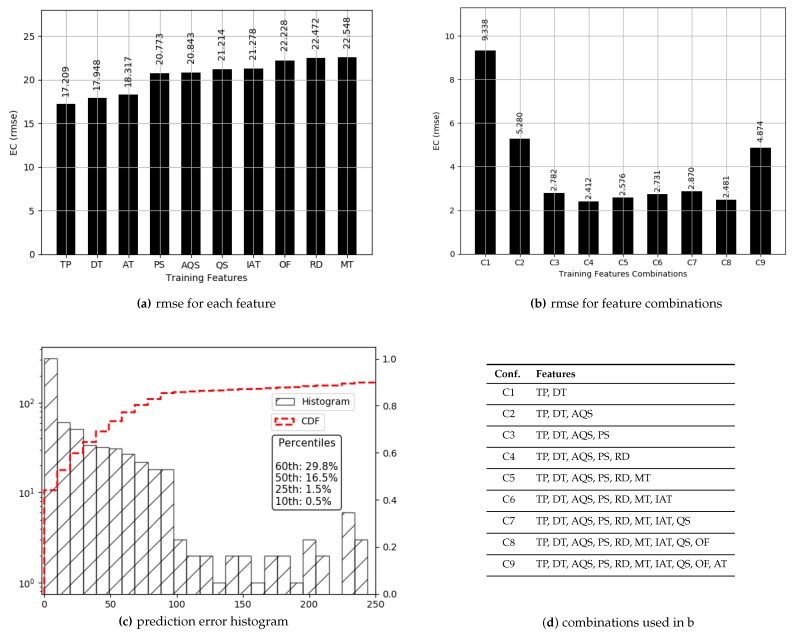
Prediction results for ECsparse.

Prediction results are described in Figure 10 and Figure 11. True values and predicted values are plotted in Figure 10, which seem to lie in a close proximity to the ideal diagonal line. However, close to the origin, there is a visible variation. To improve the understanding and to have a closer look, predicted values are plotted against actual values in Figure 11. It is visible that the majority of the predicted values are aligned with the true values. Further outcomes are presented in Figure 12. According to Figure 12a, the order of features in yielding lesser RMSE is TP, DT, AT, and PS. As against ECdense, PS moves to the fourth position and each of the next three features slide a position back. This is due to the fact that more failed transmission attempts cause EC to go up due to the failure of delivery, resulting in an enormous rise in EC per data bit, thus superseding the influence of PS. Overall, RMSE reaches its minimum (2.412) with five features. Figure 12b consists of AT, TP, PS, DT, and RD, resulting in an accuracy of 88.5% against a mean (21.09) of ECsparse. The *x*-axis label codes used in Figure 12b are explained in Figure 12d. The prediction error is characterized in Figure 12c, where 60% of the predictions have an error of 29.8% or less of the actual ECdense values. It can be conjectured that deviation, as well as lack of enough data, both contribute to this higher prediction error in ECsparse split.

It turns out that TP,DT,AT and OF are the most prominent and common features that significantly contribute to minimizing prediction error for both PDR and EC. Therefore, it encourages the combined consideration of both metrics when it comes to predicting their values. Table 4 presents the values of MPE, MAPE, Pearson Coefficient (R) and the *p*-value. It appears that the values for MPE and MAPE are very low with a close correlation represented by R. *p*-value for all predicted metrics is extremely low. These statistics further strengthen the results and encourage the adoption of predictive modeling of QoS metrics based on configurable stack parameters. Furthermore, this kind of adaptive model is sustainable because learning is based on the values of different communication stack parameters. This is in contrast to the client side approaches, where missing values in the metrics of interest are predicted without paying any regard to the inputs.

A comparison of RMSE for different regression models is presented in Table 5. It is clear that deep NN outperform all other models including baseline NN (having single layer). The only model that performs close to deep NN is random forests because of its inherent design for nonlinear data separation. From this work, it can be concluded that deep learning captures the relationships between the input parameters and performance metrics. This implies that for any change in the parameters, be it variation in value or addition of new parameters, deep learning has the potential to adjust the learning process, and the result will be a system intrinsically sustainable as it can adapt to these changes.

## 5. Conclusions and Future Work

In this work, machine learning is adopted to predict reliability in the form of PDR, and EC. A public dataset containing performance measurements for more than 48,000 combinations of different stack parameters’ (including OF, AQS, AT, IAT, TP, QS, PS, DT, RD, and MT, etc.) configurations is used to evaluate the prediction accuracy of different regression models. It turned out that deep learning performed well enough to grasp the relationship between these parameters and target metrics, and achieved an accuracy of up to 98%. It strengthens the fact that deep learning has a significant potential for performance predictions in wireless scenarios (IEEE 802.15.4 in this case). Certain features like *TP*, *DT*, *AT*, *OF*, and *PS* contributed significantly in prediction accuracy. Because many features in predicting PDR and EC are common, it can be concluded that both PDR and EC should be jointly considered. With these prediction results, deep learning offers a more practical solution compared to the legacy NP-hard optimization problems. Moreover, the computational load is on the server-side, thus easing the resource-constrained user devices. This study also vitalizes the importance of deep learning in predicting other performance metrics, and to design a comprehensive QoS solution for WSNs and the IoT, which is difficult to realize using conventional mathematical approaches. In the future, the surge in this domain will be broadened by including parameters like MAC and routing protocols, number of nodes, topology, and interference, etc. Including more variables will potentially help the learning process in better understanding the relationship between those variables and performance metrics, thus potentially yielding even better prediction accuracy and more general solutions. The aim is to collect comprehensive datasets under diverse scenarios to extend research in this direction. 

## Figures and Tables

**Table 1:** Literature survey.

Ref.	Objective	Metrics	Inputs	Approach	Evaluation	Computation	Dataset	Domain
[19]	predict the probability of success of delivery of next packet	-	PRR,SNR RSSI,LQI	NB LogR NN	MSE Delivery Cost	Server	generated using MoteLab [20]	WSN
[21]	missing QoS values pred-iction under various matrix densities and learning algorithm dimensionalities	RT THP	-	MF	Actaul vs Predicted values	Client	public dataset from Zheng [22]	IoT
[23]	missing QoS values pred-iction under various matrix densities and learning algorithm dimensionalities	RT, THP	-	CloudPred EMF,PMF LN_LFM NMF)	MAE RMSE	Client	public dataset from Zheng [22]	IoT
[24]	predictable network perf-ormance in terms of reliability	PLR	DT,IPI rP,errP	NN	RMSE	Server	generated using w.ilab-t testbed [25]	WSN
[26]	missing QoS values pred-iction under various matrix densities and learning algorithm dimensionalities	RT THP	-	CSMF	MAE NMAE RMSE	Client	public dataset from Zheng [22]	IoT/Cloud
[27]	missing QoS values pred-iction under various matrix densities and learning algorithm dimensionalities	RT	-	persistence ARIMA GRU LSTM	RMSE	Client	public dataset from Cavallo [28] and self-generated dataset using Raspberry Pi and Arduino [29]	IoT
[8]	missing QoS values pred-iction under various matrix densities and learning algorithm dimensionalities	RT THP	-	PCC Kendall’s Tau	MAE MRE RMSE	Client	public dataset from Zheng [22]	IoT
[30]	missing QoS values pred-iction under various matrix densities and learning algorithm dimensionalities	RT THP	-	DNM	MAE RMSE NMAE	Client	public dataset from Zheng [22] and extension from Tang [31]	IoT/Cloud
[32]	predictable network perf-ormance in terms of lifet-ime, transmission power level and distance	LT,TP DT	GS,TP DT,LT	NN	RMSE MAPE MPE,R T-test	Server	generated using simulations in MATLAB	WSN
This work	predictable network perf-ormance in terms of relia-bility and energy in relati-on to more than 48,000 configurations of stack parameters	PDR, EC	IAT,PS QS,MT RD,TP DT,OF AQS,AT	deep learning	RMSE MPE MAP R	Server	public dataset from Fu [33]	IoT/WSN

**Table 2:** List of acronyms and symbols used in this text.

Acronym	Stands for	Acronym	Stands for
AQS	Actual Queue Size	MRE	Mean Relative Error
ARIMA	AutoRegressive Integrated Moving Average	MT	Maximum Transmissions
AT	Actual Transmissions	NB	Naive Bayes
CSMF	Context Sensitive Matrix Factorization	NMAE	Normalized Mean Absolute Error
DL	Deep Learning	NMF	Nor-negative Matrix Factorization
DNM	Deep Neural Model	NN	Neural Networks
DT	Distance	NoN	Number of Nodes
EC	Energy Concumption	OF	Overflow
EMF	Extended Matrix Factorization	PCC	Pearson’s Correlation Coefficient
errP	Erroneous Packets	PDR	Packet Delivery Ratio
GRU	Gated Recurrent Units	PLR	Packet Loss Ratio
GS	Grid Size	PMF	Personalized Matrix Factorization
HVAC	Heating, Ventilation, Air-Conditioning	PRR	Packet Reception Ratio
IAT	Inter Arrival Time	PS	Packet Size
IoT	Internet of Things	QoS	Quality of Service
IPI	Inter Packet Interval	QS	Queue Size
LN_LMF	Latent Factor Models	R	Pearson’s Coefficient
LQI	Link Quality Indicator	RD	Retry Delay
LR	Logistic Regression	RMSE	Root Mean Square Error
LSTM	Long Short Term Memory	rP	Received Packets
LT	Lifetime	RSSI	Received Signal Strength Indicator
MAC	Medium Access Control	RT	Response Time
MAE	Mean Absolute Error	SNR	Signal-to-Noise Ratio
MAPE	Mean Absolute Percentage Error	THP	Throughput
MF	Matrix Factorization	TP	Transmission Power
ML	Machine Learning	WSNs	Wireless Sensor Networks
MPE	Mean Percentage Error		
**Symbol**	**Description**	**Symbol**	**Description**
α	Learning rate	$m_{t}$	Number of tuples in test data
$A^{{}^{\left[l\right]}}$	Result of $g^{{}^{\left[l\right]}}\left(Z^{{}^{\left[l\right]}}\right)$	$PDR_{med}$	Median of packet delivery ratio
$A^{{}^{[l].T}}$	Transpose of $A^{{}^{\left[l\right]}}$	$PDR_{std}$	Standard deviation of packet delivery ratio
$b^{{}^{\left[l\right]}}$	Regression constant at layer *l*	$PDR_{T}$	Ratio of $Pkts_{acked}$ and $Pkts_{att}$
$dA^{{}^{\left[l\right]}}$	Derivative of $A^{{}^{\left[l\right]}}$	$Pkts_{acked}$	Number of packets acknowledged
$db^{{}^{\left[l\right]}}$	Derivative of $b^{{}^{\left[l\right]}}$	$Pkts_{att}$	Actual number of transmission attempts
$dW^{{}^{\left[l\right]}}$	Derivative of $W^{{}^{\left[l\right]}}$	$Pkts_{tot}$	Total packets transmitted
$dZ^{{}^{\left[l\right]}}$	Derivative of $Z^{{}^{\left[l\right]}}$	$W^{{}^{\left[l\right]}}$	Weights assigned to inputs at layer *l*
*D*	Payload size (bytes)	*Y*	Actual outputlabels vector
$err_{k}$	Difference of $Y_{k}^{actual}$ and $Y_{k}^{predicted}$	$Y_{k}^{actual}$	Actual output of $k^{th}$ tuple
$g^{{}^{\left[l\right]}}$	Activation function at layer *l*	$Y_{k}^{predicted}$	Predicted value of $k^{th}$ tuple
$g^{{}^{{\left[l\right]}^{\prime }}}$	Derivative of $g^{{}^{\left[l\right]}}$	$Z^{{}^{\left[l\right]}}$	Output of layer *l*
*H*	Size of headers (bytes)	$z_{score}$	Value 3 deviations away from $PDR_{med}$
*m*	Number of tuples in training data		

**Table 3:** Stack parameter configurations.

Layer	Parameters	Values
Application	Inter-Arrival Time: IAT (ms)	10, 15, 20, 25, 30, 35, 40, 50
	Packet Size: PS (bytes)	20, 35, 50, 65, 80, 95, 110
Medium Access Control	maximum Queue Size: QS	1, 30, 60
	Actual Queue Size: AQS	actual values (0–60)
	buffer OverFlow: OF	actual values (0–1)
	Maximum Transmission: MT	1, 3, 5
	Actual Transmission attempt: AT	actual values (0–5)
	Retry Delay: RD (ms)	30, 60
Physical	Transmission Power level: TP	3, 7, 11, 15, 19, 23, 27, 31
	Distance: DT	10, 15, 20, 25, 30, 35

**Table 4:** Prediction performance statistics.

Metric	RMSE	MPE	MAPE	R	*p*-Value
PDR	0.012	−0.035327	0.035450	0.986	<0.001
ECdense	0.006	−0.000008	0.000192	0.968	<0.001
ECsparse	2.412	−0.128274	0.163637	0.966	<0.001

**Table 5:** RMSE of different machine learning models.

Target	Linear Regression	Gradient Boosting	Random Forest	Baseline NN	Deep NN
PDR	0.124	0.053	0.014	0.039	0.012
ECdense	0.052	0.028	0.007	0.026	0.006
ECsparse	19.21	6.423	3.017	7.924	2.412
